# A cluster randomized controlled platform trial comparing group MEmory specificity training (MEST) to group psychoeducation and supportive counselling (PSC) in the treatment of recurrent depression

**DOI:** 10.1016/j.brat.2018.03.004

**Published:** 2018-06

**Authors:** Aliza Werner-Seidler, Caitlin Hitchcock, Anna Bevan, Anna McKinnon, Julia Gillard, Theresa Dahm, Isobel Chadwick, Inderpal Panesar, Lauren Breakwell, Viola Mueller, Evangeline Rodrigues, Catrin Rees, Siobhan Gormley, Susanne Schweizer, Peter Watson, Filip Raes, Laura Jobson, Tim Dalgleish

**Affiliations:** a Medical Research Council Cognition and Brain Sciences Unit, University of Cambridge, UK; b Black Dog Institute, University of New South Wales, Sydney, Australia; c Cambridgeshire and Peterborough NHS Foundation Trust, UK; d Faculty of Psychology and Educational Sciences, University of Leuven, Belgium; e Medicine, Nursing and Health Sciences, Monash University, Sydney, Australia

**Keywords:** Depression, Memory specificity training, Autobiographical memory

## Abstract

Impaired ability to recall specific autobiographical memories is characteristic of depression, which when reversed, may have therapeutic benefits. This cluster-randomized controlled pilot trial investigated efficacy and aspects of acceptability, and feasibility of MEmory Specificity Training (MEST) relative to Psychoeducation and Supportive Counselling (PSC) for Major Depressive Disorder (N = 62). A key aim of this study was to determine a range of effect size estimates to inform a later phase trial. Assessments were completed at baseline, post-treatment and 3-month follow-up. The cognitive process outcome was memory specificity. The primary clinical outcome was symptoms on the Beck Depression Inventory-II at 3-month follow-up. The MEST group demonstrated greater improvement in memory specificity relative to PSC at post-intervention (d = 0.88) and follow-up (d = 0.74), relative to PSC. Both groups experienced a reduction in depressive symptoms at 3-month follow-up (d = 0.67). However, there was no support for a greater improvement in depressive symptoms at 3 months following MEST relative to PSC (d = −0.04). Although MEST generated changes on memory specificity and improved depressive symptoms, results provide no indication that MEST is superior to PSC in the resolution of self-reported depressive symptoms. Implications for later-phase definitive trials of MEST are discussed.

## Highlights

• Memory specificity training (MEST) was compared to supportive counselling.
• MEST produced large effects on memory specificity and depressive symptoms.
• There was no between-group difference in depressive outcomes at post or follow-up.

## 1 Introduction

Psychological treatments for depression are effective, yet there is considerable scope to improve their accessibility. Psychological interventions such as cognitive-behavioral therapy (CBT) or structured, supportive counselling can be difficult to access due to availability and cost. A shortage of skilled mental health professionals who are trained to deliver these interventions means that 54% of those who require immediate psychological intervention will spend approximately three months on a waiting list, with 12% waiting more than one year before these interventions are received (Docherty & Thornicroft, 2015; Mind, 2013). Depression is the leading cause of disability worldwide (WHO, 2017) and if it is to be better treated, the availability of effective treatments must be improved. Therefore, further investigation is needed into novel ‘low intensity’ psychological approaches which can be delivered by non-specialists and are thereby relatively inexpensive, and can be easily scaled.

There has been accumulating interest in autobiographical memory training procedures as one such option for a range of psychological disorders, including depression (Hitchcock, Werner-Seidler, Blackwell, & Dalgleish, 2017). Memory training in depression focuses on reversing memory biases that are characteristic of the disorder in order to bring about improvements in symptoms, and considerable research (for review see Hitchcock et al., 2017) has focussed on reducing overgeneral retrieval of autobiographical memory (OGM). It is well established that depression is associated with pervasive difficulties in retrieving highly specific memories of past personal events when prompted (e.g. *I enjoyed John's party last autumn*). OGM refers to the tendency for depressed individuals instead to provide summary categories of repeated events (e.g., *I always drink too much at parties*), or extended memories of events that lasted for a protracted period of time (e.g., *Last autumn was a difficult time for me at work*) rather than the required specific memories of discrete occurrences (Williams et al., 2007). The CaR-FA-X model outlines three mechanisms which underpin the proclivity to recall overgeneral memories in depression; Capture and Rumination, Functional Avoidance, and impaired eXecutive control (Williams et al., 2007). The model suggests that during the search for a specific memory of an event, attention can be captured by depression-relevant material that is general in nature, triggering rumination and therefore interrupting the search and preventing the retrieval of the specific information. The second factor, functional avoidance, proposes that OGM is an avoidance strategy that serves to mitigate the unwanted affective impact of recalling specific memories that contain emotionally-distressing material. The final aspect of the model, executive dysfunction, proposes that the availability of mental resources required to remain focused on the memory retrieval task is compromised in depression. There is empirical support for each component of the model (e.g., Dalgleish et al., 2007; Hermans et al., 2008; Watkins & Teasdale, 2001).

OGM causes impairment in everyday functioning due to its association with a range of dysfunctional processes including difficulty imagining future events (Jing, Madore, & Schacter, 2016), impaired capacity for problem solving (Goddard, Dritschel, & Burton, 1996), and difficulties with the sharing of personal material (Beike, Brandon, & Cole, 2016). Disturbances across these domains appear to have an ongoing negative impact on planning for the future, goal-directed activity, interpersonal relationships, and subsequently low mood (Williams et al., 2007). Poor accessibility of specific memories drives the unrestrained, overgeneralised negative self-judgements which contribute to worthlessness and hopelessness (Hitchcock, Rees, & Dalgleish, 2017). Further, the inability to recall emotionally vivid, specific memories of positive experiences both exacerbates anhedonia and reduces the ability to repair transient low mood (Joormann & Siemer, 2004). It is therefore not surprising that OGM is a strong predictor of the course and chronicity of major depression over and above current symptom levels (Brittlebank, Scott, Williams, & Ferrier, 1993; Sumner, Griffith, & Mineka, 2010).

Compromised memory specificity thereby represents a logical and attractive target for therapeutic intervention. Memory specificity appears modifiable (Jing et al., 2016; Yeung, Dalgleish, Golden, & Schartau, 2006); consequently, interventions that improve access to specific memories should, in theory, provide therapeutic benefit by ameliorating the adverse consequences associated with OGM (Hitchcock et al., 2017). Initial support for this hypothesis came from the finding that a brief (4 session), group-based psychological intervention – MEmory Specificity Training (MEST) – was successful in reducing OGM and ameliorating a number of associated cognitive processes in depressed individuals in an uncontrolled clinical trial (Raes, Williams, & Hermans, 2009). Training simply involved practice in retrieving specific memories to a range of cue words, delivered across four, weekly sessions and complemented by between-session homework. Using a pre-post design, results indicated that participants were more specific in memory retrieval after completing the training, which was accompanied by improvements in problem solving, rumination, and hopelessness (Raes et al., 2009). Although this trial was uncontrolled and thus limited by several design factors, it nonetheless confirmed that, in principle, memory specificity can be trained. A subsequent single-group pre-post pilot study investigated the feasibility of delivering MEST in an outpatient setting, and replicated the previous findings of increased specificity and decreased depressive symptoms following training (Eigenhuis, Seldenrijk, van Schaik, Raes, & van Oppen, 2017). Moreover, participants were encouraging of the intervention content and therapists supported the minimal training required to deliver MEST. These results suggest that the MEST programme is a feasible approach that can be delivered in an outpatient setting and could be an efficacious intervention for depression.

A follow-up pilot randomized controlled trial compared the MEST programme to a no-treatment control condition in a sample of 23 bereaved, depressed Afghani teenagers living in Iran (Neshat-Doost et al., 2013). Replicating findings from the initial uncontrolled study, MEST improved memory specificity in the experimental group, while specificity did not change in the control group. Although there were no symptom differences between conditions immediately following the programme, at two-month follow-up the MEST group also reported fewer depressive symptoms than the control group. Notably, changes in memory specificity from pre-to post-intervention mediated the reduction in depressive symptoms at follow-up suggesting that memory specificity was a mechanism involved in the improvement in symptoms.

These preliminary studies support the hypothesis that MEST produces benefits in terms of memory specificity, with the latter study also suggesting an effect on depressive symptoms. In accordance with Medical Research Council guidelines (Craig et al., 2011), once early phase studies have established a foundation of evidence, larger scale pilot trials, or platform trials, against an active intervention are needed to more comprehensively evaluate the intervention. Pilot trials of this nature ensure the recruitment, assessment, and treatment components run smoothly, and provide a platform for progression to a fully-powered, definitive trial (National Institute for Health Research [NIHR], 2017). The current study therefore used a blinded, randomized controlled design to compare MEST to an active intervention – a group psychoeducation and supportive counselling (PSC) program. The administered PSC protocol was derived from a Rogerian humanistic, non-directive supportive counselling framework (Cooper, O'Hara, Schmid, & Wyatt, 2007) and combined general psychoeducation about depression with counselling and support provided by both the group facilitators and peers. Both supportive counselling and peer support interventions are regarded as effective for the treatment of adult depression (Cape, Whittington, Buszewicz, Wallace, & Underwood, 2010; Gibbard & Hanley, 2008; Jacobs & Reupert, 2014; Pfeiffer, Heisler, Piette, Rogers, & Valenstein, 2011), with meta-analyses indicating effect sizes between 0.32 and 0.58 relative to usual treatment and non-treatment control groups (Cape et al., 2010; Cuijpers et al., 2012). The inclusion of an active comparison condition represents an important advance on previous MEST studies because it will allow for the range of likely effect sizes of MEST relative to PSC to be estimated, which can inform design for a later-phase fully powered trial.

As this was a pilot, platform trial the sample size target (*N* = 60) was set to allow us estimate the range of likely effect sizes delivered by MEST, relative to PSC (see the trial protocol; Dalgleish et al., 2014). This contrasts with a fully-powered definitive trial where the likely effect sizes have been previously established and sample sizes are set to provide the statistical power to reject the null hypothesis of no difference between treatment arms (MRC, 2000). The findings from this trial can then provide appropriate effect-size estimates for MEST that can be used for sample-size calculations in later fully-powered definitive trials (Lancaster, Dodd, & Williamson, 2004).

If MEST turns out to be efficacious it would offer an attractive, accessible therapeutic option for depression as it is brief (only 5 weeks) and can be delivered by individuals who have not had as extensive training as that required for more complex psychological interventions such as supportive counselling or cognitive behaviour therapy. This is due to a detailed prescriptive training manual which includes both therapist and client materials for each session. The treatment does not require the therapist to challenge or confront maladaptive thinking, facilitate problem solving, provide counselling, or draw links between, or reconcile, the past experiences of participants. This makes MEST an appealing candidate as a low-intensity programme that can be readily scaled, and is cheaper and easier to deliver than current psychological interventions which require considerable training in a variety of therapeutic techniques.

The primary endpoint for this trial was self-reported depressive symptoms at three months post-treatment following MEST, as compared to PSC. Secondary outcomes were depressive symptoms at post-intervention, and diagnostic status, depression-free days and memory specificity at both post and 3-month follow-up, along with diagnostic status and depression-free days at 6-months for the MEST group only (as the PSC group were offered MEST at the 3-month point). We also explored whether any reduction in depressive symptoms was mediated by change in memory specificity (cf. Neshat-Doost et al., 2013; see the published trial protocol for details; Dalgleish et al., 2014).

## 2 Method

This trial was registered at clinicaltrials.gov (#NCT01882452). The full protocol was published (Dalgleish et al., 2014).

### 2.1 Trial design

We completed a single-blind, parallel cluster randomized controlled trial (RCT) comparing MEST to a psychoeducation and supportive counselling intervention, with an embedded exploratory mechanisms study. Assessments were conducted at baseline, post-intervention, and 3-month follow-up in both arms, with an additional 6-month follow-up assessment for the MEST group only. The primary outcome was self-reported depression at 3 months follow-up.

### 2.2 Participants

As noted in the Introduction, although a standard power calculation based on detecting treatment effects would be the conventional approach to determining sample size for a definitive clinical trial, the main aim of the current pilot trial was to investigate the likely efficacy of MEST, in preparation for scaled-up later phase fully-powered evaluations of the intervention in line with MRC guidance (MRC, 2000). We deemed that 60 participants (30 in each arm) would provide sufficient numbers to estimate efficacy, provide an initial indication of feasibility of the trial procedures and acceptability of the intervention, and to generate preliminary process data for this treatment. This sample size would provide 24 patients in each arm, allowing for 20% attrition. This would provide a plausible range of point estimates of effect on our set of outcome measures sufficient to guide us in sample size calculations for later phase trial work (Dalgleish et al., 2014).

Inclusion criteria were a diagnosis of Major Depressive Disorder (MDD; recurrent) according to the Structured Clinical Interview for the DSM-IV Axis I Disorders - Clinician Version (First, Spitzer, Gibbon, & Williams, 1995), a diagnosis of a current Major Depressive Episode (MDE), together with the presence of moderate symptoms defined on the Beck Depression Inventory II as > 13 (BDI-II; Beck, Steer, & Brown, 1996), and a level of memory specificity of lower than 70% as assessed on the Autobiographical Memory Test (AMT; Williams & Broadbent, 1986) during the screening session (see Procedures). A deficit in memory specificity was a necessary inclusion criterion because, unless specificity is compromised, there is little opportunity for benefits to accrue. SCIDs were completed by trained research staff under the supervision of a clinical psychologist (AWS or CH). Scoring on 100% of SCIDs was second-rated by the clinical psychologist, which resulted in 100% agreement on diagnostic status for primary and comorbid disorders. Exclusion criteria were high levels of suicidal intent (ascertained from the clinical interview and based on clinical judgement), current symptoms of psychosis or current drug or alcohol abuse or dependence (all assessed via the SCID-IV), a diagnosed Axis II disorder (assessed via participant report), or the presence of intellectual disability or traumatic brain injury (also verified by participant report).

Participants were recruited in three ways. The first pathway was direct from the community using online and print advertisements. Interested participants were screened over the phone for previous mental health problems, current mood difficulties, and brain injury. Suitable participants were invited into the laboratory to verify study eligibility. The second pathway was through clinical services offered within the Cambridgeshire and Peterborough NHS Foundation Trust, UK. Service-users were identified by the clinical team and provided with an information sheet about the trial. These individuals opted in by contacting the research team and were then screened for eligibility as already described. The final pathway was from our departmental mental health panel. These individuals had previously responded to advertisements requesting volunteers with depression to assist with research. Again, individuals were screened over the phone and suitable participants were invited in for a screening session to verify eligibility.

### 2.3 Interventions

#### 2.3.1 MEmory specificity training (MEST)

The original treatment manual (Raes et al., 2009) was translated from Dutch into English, and was extended from four to five sessions, in line with the initial pilot RCT (Neshat-Doost et al., 2013). The manualized programme involved two therapists delivering treatment over 5 × 60 min sessions to groups of 5–8 individuals. Between sessions, participants were required to keep a diary in which they recorded a specific memory of the day, and completed further cued-recall exercises.

Over the five sessions, participants practiced recalling specific memories in response to a range of different cues (words and pictures of different emotional valence), with weekly homework. Session 1 involved psychoeducation about memory difficulties common in depression and practice recalling specific memories in response to positive and neutral cues. Session 2 followed the same format with further practice. In Session 3, memory retrieval using negative cues was introduced. Session 4 involved practice to positive, negative, and neutral cues as well as a discussion of incidents in which participants noticed overgeneral thinking in everyday contexts. Session 5 involved further practice using all cue types, revision of the material covered throughout the treatment, and discussion of how to maintain what had been learnt during MEST.

#### 2.3.2 Psychoeducation and supportive counselling (PSC)

The PSC intervention followed the same format and length as the MEST intervention (i.e., delivered by two therapists to groups of 5–8 individuals over 5 × 60 min sessions). The PSC manual was developed and manualized for this trial by two clinical psychologists (AWS, TD), in consultation with two experienced counsellors from the Cambridge University Counselling Service.

A non-judgemental, supportive environment was created whereby participants were encouraged to discuss events and topics of their choice. Through reflection and discussion, the aim was to support members to better cope with everyday life. This approach employed non-directive methods (such as drawing upon the therapeutic relationship, encouraging input from group members, reflective listening), and avoided directive methods such as those taught in cognitive behavioral therapy (e.g., cognitive restructuring). To optimize the comparability between conditions, participants also kept a diary in which they were asked to record material from each day that they may wish to raise in the group. This material could involve positive, negative, or neutral daily events or themes. In Session 1, psychoeducation about depressive symptoms was presented and the experience of group members was normalized. Participants were invited to discuss what they would like to get out of attending the group. An outline of the programme was then provided and the homework tasks were introduced. Session 2 involved reviewing the homework and continued discussion of events that participants had noted down in their diaries, with therapists encouraging input from other group members. Sessions 3, 4, and 5, followed an identical structure – a homework review, followed by discussion, with the homework task to record daily events or themes for later discussion.

### 2.4 Treatment fidelity

After each session, therapists completed a Treatment Fidelity Checklist which was developed specifically for this study and measured compliance to the protocol. Fifteen percent of the audio-taped treatment sessions (half from MEST and half from PSC) were randomly selected and rated for adherence to the manual by an experienced clinician (LJ), who was not involved in the day-to-day running of the trial. Ratings (0–1) indicated that there was exceptionally high clinician adherence to the manual (adherence = 0.996).

### 2.5 Randomization and sequence generation

Participants were cluster randomized using a blocked procedure with 5–8 participants per group using computer-generated quasi-random numbers. Randomization was conducted at the start of the trial by the trial statistician (PW) and communicated to trial managers (AWS, CH) after participant groups had been recruited into the study.

### 2.6 Blinding

This was a single-blind study. All assessors and data analysts were blind to participant condition. Participants were blind to condition until the first treatment session where they were informed of their group allocation.

### 2.7 Outcome measures

#### 2.7.1 Memory specificity

Generated memories on the Autobiographical Memory Test were audiotaped for later coding and the number of specific memories was calculated. Assessors coded memories and a second-rater assessed a randomly selected 15% of these tapes. Inter-rater reliability for specificity vs non-specificity was high (κ = 0.99). We evaluated changes in memory specificity from baseline to post-intervention and to three-month follow-up, across groups.

#### 2.7.2 Primary clinical outcome

The BDI-II (Beck et al., 1996) is a 21-item measure of depressive symptoms and severity over the past two weeks, and has strong psychometric properties. The primary endpoint was three-month follow-up.

#### 2.7.3 Secondary clinical outcomes

Secondary outcomes were depressive symptoms on the BDI-II at post-intervention, and diagnostic status and depression-free days, at post-test and follow-up. Diagnostic status and depression-free days were measured using the Longitudinal Interview Follow-up Evaluation (LIFE; First et al., 1995) to the SCID. Autobiographical memory specificity was measured using the Autobiographical Memory Test (Williams & Broadbent, 1986), as employed longitudinally by Brittlebank et al. (1993). Generated memories were audiotaped for later coding and the number of specific memories was calculated. Assessors coded memories and a second-rater assessed a randomly selected 15% of these tapes. Inter-rater reliability for specificity vs non-specificity was high (κ = 0.99).

#### 2.7.4 Additional process outcomes

Process variables were measured to identify potential mechanisms of change. Problem-solving was measured using the Means Ends Problem Solving Task (MEPS; Platt & Spivack, 1975), which indexes the number of steps used to solve a problem (means) and an overall effectiveness score out of 7 (Marx, Williams, & Claridge, 1992). A greater number of means and higher effectiveness score reflect greater levels of problem-solving ability. We administered a shortened version of the MEPS (Scenarios 2, 4, 8, and 10) which is commonly used with depressed samples (Watkins & Moulds, 2005). Rumination was measured using the Ruminative Response Scale (RRS; Nolen-Hoeksema & Morrow, 1991). Cognitive avoidance was measured using the Cognitive Avoidance Questionnaire (CAQ; Sexton & Dugas, 2008), which is a 25-item measure of the degree to which an individual engages in five different cognitive avoidance strategies (thought suppression, thought substitution, distraction, avoidance of threatening stimuli, and transformation of images into thoughts). Executive function was measured using the Controlled Oral Word Association Test (COWAT) to test verbal fluency (VF), in which participants generate words in 60 s, beginning with a particular letter (i.e., F, A or Spreen & Strauss, 1998). Digit Span (DS) also tested executive function and is a subtest from the Weschler Adult Intelligence Scale (Wechsler, 2014). Short term memory was measured at baseline using the Verbal-Paired Associates task (VPA) from the Weschler Memory Scale (Wechsler, 2009), to ensure that groups did not differ on general memory functioning at baseline.

### 2.8 Procedure

Ethical approval was obtained from the UK National NHS Research Ethics Service (East of England, 11/H0305/1). Recruitment began in February 2014 and closed in April 2016. Prior to study entry, participants received an information sheet and provided written informed consent before being screened for eligibility. The screening session involved administration of the SCID-IV, the AMT and BDI-II. Eligible participants were provided detailed study information and signed another consent form in order to proceed into the trial.

Participants were tested individually in a quiet testing room by researchers blind to group status on three occasions: at baseline, post-treatment, and 3-month follow-up. The baseline assessment session consisted of the AMT, MEPS, VF, DS, VPA, along with a questionnaire package containing basic demographics, BDI-II, CAQ, and RRS. When a minimum of five participants met inclusion criteria, the group was cluster-randomized to either MEST or PSC. Following treatment, participants were re-assessed (post) on all measures, in addition to measuring the number of depression free days experienced since the beginning of the intervention using the LIFE. All measures were re-administered at three month follow-up. Task and questionnaire order, MEPS scenarios and AMT wordlists were counterbalanced across assessment sessions. Assessment sessions lasted between 45 and 60 min and participants were reimbursed at a rate of £6/hour for their time. A 6-month follow-up occurred via phone with participants allocated to the MEST group only, during which participants completed the SCID-IV Mood Module, number of depression free days on the LIFE, and returned a completed BDI-II to the experimenter. No adverse events were reported during the trial.

## 3 Results

### 3.1 Sample characteristics

**Fig. 1:**
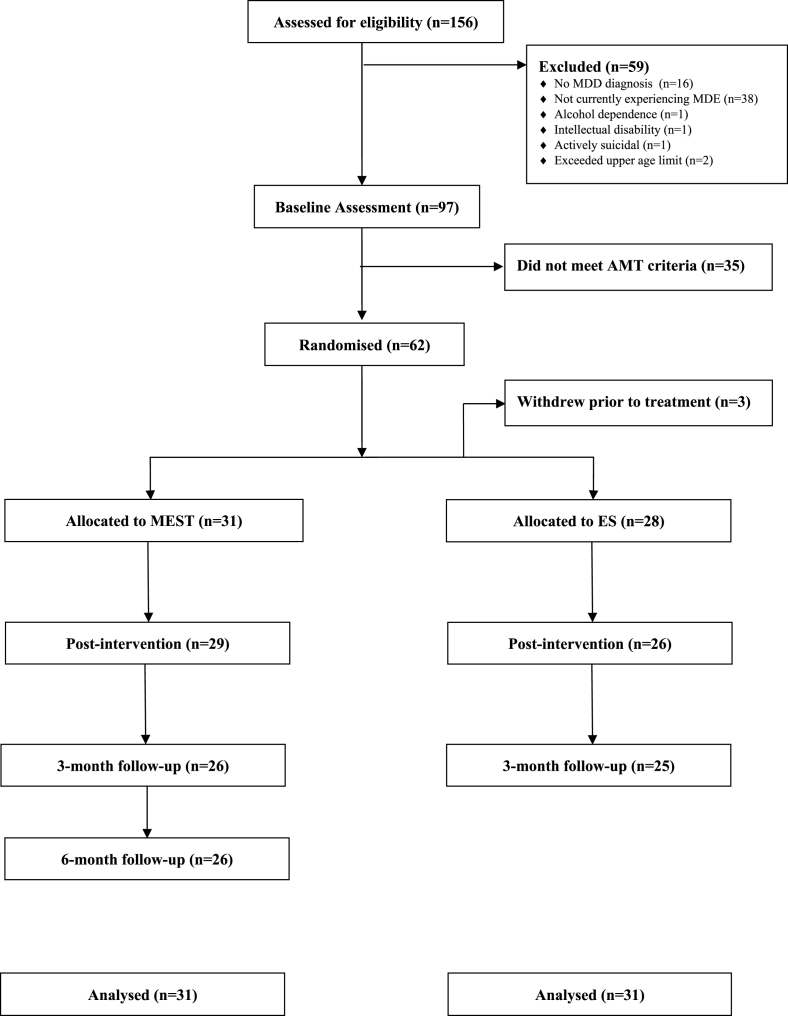
CONSORT flow chart.

Of the 156 participants assessed for eligibility, 62 met inclusion criteria. Participants were cluster randomized to either MEST (*n* = 31) or PSC (*n* = 31). The CONSORT flow chart outlines recruitment, randomization, and completion (see Fig. 1). Sample characteristics for randomized participants are shown in Table 1. The mean age of the sample was 41.77 years (*SD* = 13.6), and 66% of participants were female. Approximately half of the sample were university educated and employed at the time of the study. Sixty percent reported taking medication for depression, and 25% were receiving psychological treatment. All had a history of recurrent depression. Twenty seven participants (43%) had experienced too many prior depressive episodes to distinguish the exact number (coded as ‘too many to count’ on the SCID). For those able to distinguish the number of prior episodes, the mean was 5.03 (SD = 3.05). Forty percent of participants also met criteria for a comorbid disorder (as indexed by the SCID). Current co-morbid diagnoses were generalised anxiety disorder (*n* = 14), posttraumatic stress disorder (*n* = 8), obsessive compulsive disorder (*n* = 3), social anxiety disorder (*n* = 9), specific phobia (*n* = 2), panic disorder (*n* = 4), and eating disorder (*n* = 3).

**Table 1:** Sample characteristics at baseline for the Memory Specificity Training (MEST) and Psychoeducation and Supportive Counselling (PSC) conditions.

	PSC		MEST		χ^2^	*p*
	(n = 31)		(n = 31)			
Gender (% female)	61.3%		70.1%		0.6	.592
Education^a^	3:7:10:5:6		5:7:7:11:1		6.7 b	.152
Employment (% employed)	48.4%		41.9%		0.3	.799
Taking medication (%)	61.3%		58.1%		0.7	1.00
Receiving psychotherapy (%)	29.0%		22.5%		0.3	.772

	M	(SD)	M	(SD)	*t*	*p*
Age	39.1	(11.3)	44.45	(15.3)	−1.6	.123
BDI-II	32.9	(9.7)	30.6	(11.2)	0.9	.387
Verbal paired associates recall	16.2	(9.0)	19.2	(9.0)	−1.3	.208
Digit span	16.9	(4.8)	19.1	(4.5)	−1.8	.070

a Highest level of education attained (5th form; 6th form; undergraduate; postgraduate; other).

b Fishers Exact Test; *p < .05.

At baseline, participants reported depressive symptoms in the severe range on the BDI-II and recalled an average of 5.2 specific memories out of a possible twelve memories (43% specific) on the AMT. There were no differences between the PSC and MEST conditions at baseline on demographic variables, depressive symptoms, number of prior depressive episodes, cognitive avoidance, rumination, memory specificity, problem-solving ability, working memory or short-term memory (all *p*s > .05). However, the MEST group performed better on the verbal fluency task (*p* = .03), and baseline performance was therefore covaried in all subsequent analyses in line with the trial protocol (Dalgleish et al., 2014).

### 3.2 Treatment characteristics

Attrition was 11% at post-intervention and 20% at the 3-month follow-up (the primary endpoint) in line with pre-trial estimates (Dalgleish et al., 2014). These figures include three participants (5%) who withdrew prior to attending the first treatment session (in the PSC condition). There was no differential drop-out between the groups at either post-treatment, *χ*^*2*^ (62) = 2.30, *p* = .130, or 3-month follow up, *χ*^*2*^ (62) = 0.11, *p* = 1.00. Participants completed a mean of 3.72 of the five treatment sessions (median = 5 sessions), again with no difference in the mean number of sessions attended between groups, *χ*^*2*^ (5) = 0.23, *p* = .216.

After completing the first session, participants in the MEST condition reported greater ratings for how logical the intervention seemed (*M* = 7.25, *SD* = 1.45) than the PSC participants (*M* = 5.54, *SD* = 1.82), *t* (48) = 3.66, *p* < .001, *d* = 1.04. MEST (*M* = 5.75, *SD* = 1.75) was also perceived to be more likely to be successful in reducing symptoms than PSC (*M* = 4.27, *SD* = 20.1), *t* (48) = 2.77, *p* = .01, *d* = 0.78. There was no significant difference between the interventions in how confident participants were in recommending the program to a friend, *t* (48) = 0.86, *p* = .396, *d* = 0.24, or in how much improvement participants expected in their depressive symptoms, *t* (48) = 0.43, *p* = .673, *d* = 0.12.

### 3.3 Analysis of trial outcomes

All analyses were initially completed on an intent-to-treat (ITT) basis, using multiple imputation to account for missing data. Results did not vary between ITT and per protocol analyses (for participants who attended three of the five sessions). As such, only ITT analyses are reported. We completed mixed-model repeated measures (MMRM) analyses (Hamer & Simpson, 2009) with intervention type and time as fixed factors, and individual group allocation as a random effect to evaluate and accommodate clustering effects. We also covaried for verbal fluency to account for the observed difference at baseline. These analyses produced estimated marginal means and standard errors (Table 2), along with *F* values for between group contrasts at each time point, and these *F* values were used to calculate Cohen's *d*'s. Positive values demonstrate an effect in favor of the MEST condition. 95% confidence intervals are presented for the *d* for primary and secondary outcomes. As this was a pilot trial in preparation for a fully-powered, definitive trial, key outcomes of interest are the obtained effect sizes and their associated confidence intervals (which reflects the likely size and variability of treatment effects), rather than statistical significance of the comparisons (Lancaster et al., 2004; NIHR, 2017).

**Table 2:** Estimated marginal means and standard error (SE) for primary, secondary outcomes at each time point, and pre to post change in process measures by condition.

Outcome	Psychoeducation & Supportive Counselling						Memory Specificity Training					
	Baseline		Post		3 months		Baseline		Post		3 months	
	Mean	(SE)	Mean	(SE)	Mean	(SE)	Mean	(SE)	Mean	(SE)	Mean	(SE)
BDI-II depression score	32.6	(2.3)	27.04	(2.4)	24.1	(2.4)	30.8	(2.3)	25.8	(2.3)	24.7	(2.3)
AMT specific	5.2	(0.4)	5.8	(0.5)	6.6	(0.5)	5.2	(0.4)	8.0	(0.4)	8.5	(0.5)
Days depression free (proportion)	–	–	.39	(.07)	.40	(09)	–	–	.40	(.06)	.49	(.07)
Remission rate	–	–	42.3%	–	52.0%	–	–	–	34.5%	–	61.5%	–
Rumination	62.2	(12.1)	57.36	(2.51)	60.77	(4.58)	59.6	(12.7)	57.02	(2.32)	53.28	(4.30)
Cognitive avoidance	74.2	(20.4)	69.09	(4.42)	74.43	(24.01)	71.3	(20.4)	64.05	(4.16)	68.84	(25.08)
Problem solving - means	4.4	(2.6)	3.87	(0.56)	–	–	4.8	(2.2)	5.88	(0.53)	–	–
Problem solving- effectiveness	5.4	(3.2)	5.14	(0.58)	–	–	6.6	(2.9)	6.63	(0.55)	–	–
Verbal fluency	11.9	(6.0)^a^	13.67	(0.97)	12.55	(0.95)	15.1	(5.3)	15.71	(0.91)	16.97	(0.93)

*Note.* Estimated marginal means are estimated from a mixed-model with group number as a random effect (to account for clustering effects) and intervention condition and time as fixed effects, covarying for verbal fluency to account for group difference in verbal fluency at baseline. Note that verbal fluency was not covaried when analysing change in process measures.

a Verbal fluency differed between groups at baseline, *t* = -2.3, *p* = .03.

#### 3.3.1 Memory specificity

The therapeutic target of MEST is to improve memory specificity, and results demonstrated that the intervention achieved this aim (see Table 2). Analyses revealed a group by time interaction for memory specificity, *F* (1, 107.2) = 5.96, *p* = .004, *d* = 0.62. As anticipated, a large between-group effect-size indicated that participants in the MEST group demonstrated greater levels of memory specificity compared to the PSC group at both post-test, *F* (1, 127.34) = 11.90, *p* = .001, *d* = 0.88 [0.34, 1.42], and follow-up, *F* (1, 132.90) = 8.49, *p* = .004, *d* = 0.74 [0.21, 1.27]. There was a large within-group effect size for change in specificity in the MEST condition from pre-to post-test (*d* = 1.53).

#### 3.3.2 Primary clinical outcomes

**Fig. 2:**
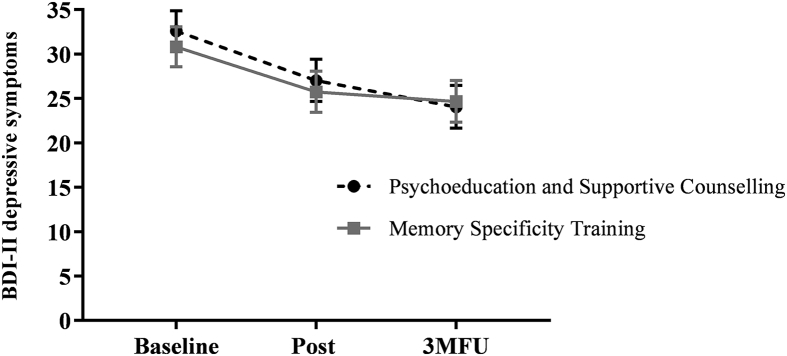
Mean (±1 SEM) depressive symptoms at post-intervention and 3-month follow-up for MEST and PSC groups.

Changes in depression characteristics are presented in Table 2 and Fig. 2. The primary outcome was BDI-II score at three month follow-up. A mixed effects model with the above specifications indicated that across groups there was a significant reduction in depressive symptoms over time, *F* (2, 102.5) = 17.5, *p* < .001, but no effect of group, *F* (1, 59.75) = 0.07, *p* = .79, nor a time × group interaction, *F* (2, 102.6) = 0.46, *p* = .63. On average, participants experienced a decrease from the severe to moderate range on the BDI-II (Beck et al., 1996) from baseline (*M* = 31.74, *SD* = 10.47) to follow-up (*M* = 23.74, *SD* = 14.14), *t* (102.8) = 3.41, *p* = .001, *d* = 0.87. However, a negligible between-group effect size was found at three month follow-up, *F* (1, 94.11) = 0.03, *p* = .86, *d* = 0.04 [-0.48, 0.56].

#### 3.3.3 Secondary clinical outcomes

Our co-secondary outcomes were BDI-II score at post-test, diagnostic status, and depression free days at post-test and three month follow-up. The mixed effects model predicting BDI-II demonstrated a small and non-significant between-group effect size in favor of the MEST group at post-test (see Table 2), *F* (1, 90.70) = 0.15, *p* = .70, *d* = 0.10, [-0.42, 0.62]. There was also a small, non-significant between-group effect size in favor of MEST in the relative number of participants who no longer met criteria for a Major Depressive Episode at both post-test, *F* (1, 89) = 0.76, *p* = .38, *d* = 0.22 [-0.34, 0.78], and follow-up, *F* (1, 89) = 0.35, *p* = .55, *d* = 0.15 [-0.43, 0.73].^2^ The 6-month follow-up assessment of the MEST group indicated that improvement in BDI-II was maintained from 3 to 6 months post-treatment (*M* = 23.26, *SD* = 13.87), *t* (18) = 1.38, *p* = .19, and that 52% of individuals no longer met criteria for a Major Depressive Episode at 6 months post-treatment.

Similarly, there was a negligible non-significant between-group effect size in favor of MEST for the difference in proportion of depression-free days experienced from pre-to post-test, *F* (1, 8) = 0.20, *p* = .66, *d* = 0.11 [-0.41, 0.63]. This was also the case for depression free days from post-test to follow-up, *F* (1, 9.11) = 0.08, *p* = .78, *d* = 0.07 [-0.44, 0.59]. At 6-months, MEST participants reported an average proportion of .55 (*SE* = 0.30) depression free days in the period since the three-month follow-up.

#### 3.3.4 Additional process outcomes

We also explored whether MEST and PSC differentially impacted the degree of change in the process variables: rumination; problem solving; cognitive avoidance; and verbal fluency (an index of executive control). A Bonferroni correction was applied to account for multiple comparisons (α = 0.05/5, corrected *p* = .01). Trivial to small (Cohen, 1992) non-significant between-group effect sizes (descriptive statistics in Table 2) were observed on pre-to post-intervention change in rumination, *F* (1, 46) = 0.14, *p* = .71, *d* = 0.11, cognitive avoidance, *F* (1, 44) = 0.05, *p* = .82, *d* = 0.06, and verbal fluency, *F* (1, 48) = 0.48, *p* = .79, *d* = 0.19. For problem solving ability, there was a moderate between-group effect size in favor of the MEST group for the number of means (or steps) used to solve a problem, *F* (1, 46) = 5.95, *p* = .02, *d* = 0.68. A trivial between-group effect size was observed for effectiveness of problem solving, *F* (1, 46) = 0.18, *p* = .67, *d* = 0.12.

### 3.4 Exploratory analyses

We next explored whether group-based change in memory specificity from pre-to post-test would underlie improvement in depressive symptoms at follow-up. This hypothesis was not supported, Δ*R*^2^ = 0.024, *F* (1, 45) = 2.05, *p* = .16. Similarly, change in specificity did not significantly impact BDI-II at follow-up (adjusting for BDI-II at pre-intervention), across all participants (*β* = −.51, *p* = .176), nor in the in the MEST group alone (*β* = 0.11, *p* = .287).

We also explored whether change in problem solving skills, rumination, cognitive avoidance, or verbal fluency mediated the effect of group on BDI-II at follow-up creating 1000 bootstrapped samples in PROCESS (Hayes, 2013). All 95% confidence intervals for indirect effects spanned zero, and thus demonstrated no significant mediations.

## 4 Discussion

The core aim of this pilot RCT was to assess the effects of MEST on our cognitive target of memory specificity and on a range of clinical measures of depression, relative to an active psychoeducation and support counselling (PSC) intervention, to provide a platform for a later definitive trial. As anticipated, MEST (but not PSC) delivered large effects for change (*d* = 0.88 at post, *d* = 0.74 at follow-up) on our target cognitive mechanism variable of memory specificity. Furthermore, on our primary clinical endpoint of self-reported depressive symptoms at three-month follow-up, both MEST and PSC led to a large decrease in symptoms from the severe range on the BDI-II on average at baseline, to the moderate range at follow-up (*d* = 0.87). Data available from the MEST participants at six-month follow-up also indicated that their improvement in depressive symptoms was maintained.

However, we found no support for MEST being a clinically superior intervention to PSC, with a negligible effect size difference between the treatment arms on our primary outcome (*d* = 0.04). Secondary depressive outcomes largely mirrored this finding, with negligible to small between-group effects in favor of MEST (*d*s from 0.07 to 0.22) for depressive symptoms at post-intervention, for the number of participants meeting criteria for MDD, and for the number of depression-free days experienced, at both post-test or three-month follow-up. Taken together, the results provide no support that MEST is likely to be superior to PSC in the amelioration of depression. That is, the data suggest that MEST does not provide any additional benefits over and above that offered by existing treatments for depression such as PSC. Although MEST delivered superior effect sizes for change in memory specificity relative to PSC, we found no support in our exploratory analyses that changes in our target process mediated clinical outcomes in the trial. This is perhaps unsurprising due to the relatively small sample size, the negligible difference between the treatment arms on the clinical endpoint, and that the current study was underpowered to detect significant group differences between conditions. Any fully-powered definitive trial would need to explore the role of specificity as a mechanism of change. The failure of memory specificity to mediate clinical outcomes suggests that the causal relationship between memory specificity and depressive symptoms may be more complex than previously indicated. Therefore, it will be important for future studies to investigate the process through which specificity influences depressive symptoms. We found no support for superior effects of MEST relative to PSC on our other process measures.

If we consider the pre-post within-arm effect size for the effects of MEST on depressive symptoms in the current study (*d* = 2.65 at three-month follow-up), we find that it is actually larger than that found in our pilot RCT (Neshat-Doost et al., 2013; *d* = 0.47 at two-month follow-up). However, the pilot trial assessed MEST against a no-intervention control condition, whereas in the current study the comparison intervention comprised an active treatment. The use of an active comparator group in controlled trials evaluating psychotherapy for depression has been found to be associated with small effect sizes and non-significant results, particularly in high quality trials involving manualized treatment, therapist training and supervision, and therapist fidelity checks (Mohr et al., 2014). Furthermore, active control groups that are matched to the experimental group in terms of treatment length, credibility and implementation often have a considerable impact on treatment outcome (Mohr et al., 2008). Therefore, while inclusion of an active comparison intervention controls for non-specific treatment factors such as contact with a supportive mental health professional, and exposure to beneficial group processes and peer support, it nonetheless makes it very challenging to show superiority of the experimental group. The inclusion of a non-active control group such as a waitlist control or treatment as usual group would have uncovered the extent to which MEST and PSC were useful in the treatment of depression in a UK healthcare context, in line with our previous small pilot trial of MEST conducted outside of the UK (Neshat-Doost et al., 2013). This represents a limitation of the current study and in order to illustrate the extent to which these active interventions are efficacious, any future trials should include both active and non-active control groups, in the same study.

The decrease in symptoms following MEST from the severe to moderate range on the BDI-II, reflecting a minimally clinically significant symptom improvement of 20% from baseline BDI scores (Button et al., 2015; Viljoen, Iverson, Griffiths, & Woodward, 2003), along with the low drop-out rate (16%) provides preliminary evidence that MEST may be useful as a low-intensity intervention. Although our findings indicate that MEST does not improve upon current treatment options for depression, it may still hold value as a programme that shifts an additional risk factor for relapse (memory specificity) that can be delivered in group format by non-specialists with minimal training. Any future work to evaluate MEST should be completed by research groups who have not been involved in the development of the programme, to address researcher allegiance, and prior to any definitive trial, more work is needed to elucidate the processes through which memory specificity impacts depressive symptoms.

The lack of support for any difference between MEST and PSC indicates that any further evaluation of MEST should investigate MEST's *non-inferiority*, rather than superiority. That is, we recommend that the next step in this line of enquiry be to conduct a powered non-inferiority trial which compares MEST to established evidence-based group treatments for depression such as PSC or CBT (Cuijpers et al., 2012; Okumura & Ichikura, 2014). PSC requires highly skilled therapists who can provide a safe and non-judgemental environment for their clients to discuss the causes of their distress. CBT also requires highly skilled therapists to deliver structured skills-based therapy which teaches clients how to challenge automatic, depressogenic patterns of thought and re-engage with the world that they have often withdrawn from. Such a trial should also include a robust health economics analysis to examine the cost-effectiveness of MEST relative to comparison interventions. If MEST were found to be more cost-effective and non-inferior in treatment effects to either of these interventions, the advantage would be that it is of lower intensity, requiring less specialised clinical training and characterized by greater simplicity in delivery.

## Trial registration

NCT01882452 (ClinicalTrials.gov).

## Protocol published

www.trialsjournal.com/content/15/1/293.

## Funding

MC_US_A060_0019, Medical Research Council, UK.

## Appendix A Supplementary data

Published protocol Published protocol

## Footnotes

2 It is possible that the use of self-report measures as our primary and secondary clinical outcomes may have influenced results due to their reliance on memory. However, the effect sizes were mirrored in the clinician-administered measure of depression status.
